# Expression of Transcript Variants of *PTGS1* and *PTGS2* Genes among Patients with Chronic Rhinosinusitis with Nasal Polyps

**DOI:** 10.3390/diagnostics11010135

**Published:** 2021-01-16

**Authors:** Wioletta Pietruszewska, Wojciech Fendler, Marta Podwysocka, Adam J. Białas, Piotr Kuna, Izabela Kupryś-Lipińska, Maciej Borowiec

**Affiliations:** 1 Department of Otolaryngology, Head and Neck Oncology, Medical University of Lodz, 90-419 Lodz, Poland; marta.podwysocka@gmail.com; 2 Department of Radiation Oncology, Dana-Farber Cancer Institute, Boston, MA 02115, USA; wojciech_fendler@dfci.harvard.edu; 3 Department of Biostatistics and Translational Medicine, Medical University of Lodz, 90-419 Lodz, Poland; 4 Department of Pathobiology of Respiratory Diseases, Chair of Internal Medicine, Medical University of Lodz, 90-419 Lodz, Poland; adam.bialas@umed.lodz.pl; 5 Department of Internal Medicine, Asthma and Allergy, Medical University of Lodz, 90-419 Lodz, Poland; piotr.kuna@umed.lodz.pl (P.K.); izabela.kuprys-lipinska@umed.lodz.pl (I.K.-L.); 6 Department of Clinical Genetics, Medical University of Lodz, 90-419 Lodz, Poland; maciej.borowiec@umed.lodz.pl

**Keywords:** chronic rhinosinusitis with nasal polyps, CRSwNP, cyclooxygenase-1, cyclooxygenase-2, NSAIDs, non-steroidal anti-inflammatory drugs, *PTGS1* gene, prostaglandin-endoperoxide synthase 1 gene, *PTGS2* gene, prostaglandin-endoperoxide synthase 2 gene, transcript variant

## Abstract

To date, there has been no reliable test to identify unfavorable course of Chronic Rhinosinusitis with Nasal Polyps (CRSwNP), especially in aspirin intolerant patients. The research aimed to analyze the expression of transcript variants of *PTGS1* and *PTGS2* genes in the pathobiology of the disease. The study was performed on 409 adult patients: 206 CRSwNP patients including 44 (21.36%) aspirin intolerant patients and 203 healthy volunteers in the control group. Transcript variants of the *PTGS1* and *PTGS2* genes named as follows: COX1.1 for NM_000962, COX1.2 for NM_080591, COX1.3 for NM_001271165.1, COX1.4 for NM_001271368.1, COX1.5 for NM_001271166.1, COX2.1 for NM_000963.3, COX2.2 for AY_151286 and COX2.3 for BQ_722004 were confirmed using direct sequencing and quantified using targeted qPCR. The coexistence of all examined transcript variants in the study and the control group and significant differences between both were found. In aspirin sensitive patients, the levels of COX1.2, COX1.3, COX1.4 and COX1.5 isoforms were higher compared to aspirin-tolerant patients. The severity of symptoms was bigger in patients with higher expressions of variants: COX1.1 (R with dCt = −0.134; *p* = 0.0490), COX1.3 (R = −0.1429; *p* = 0.0400) and COX1.5 (Rs = −0.1499; *p* = 0.032). The expression of COX1.1 (Rs = −0.098; *p* = 0.049) and COX1.5 (Rs = −0.141; *p* = 0.043) isoforms increased with polyposis advancement in endoscopy. With the CT extent of sinuses opacification, COX1.1 isoform also significantly increased (Rs = −0.163; *p* = 0.020). The isoforms COX1.3, COX1.4, COX1.5 and COX2.1 may promote milder CRSwNP course. On the contrary, the variants COX1.1, COX1.2 and COX2.2 may be involved in a more aggressive disease.

## 1. Introduction

Chronic rhinosinusitis with nasal polyps (CRSwNP) is a relapsing disease with growing incidence rate [1,2]. CRSwNP occurs in about 11% of European population, affecting 20% of patients suffering from chronic diseases of aerodigestive tract, with predominance of men over 60 years old [3,4]. Despite using proper conservative treatment, advanced surgical techniques and complementary long-term pharmacotherapy, CRSwNP is characterized by irregular and hard to predict recurrences [5]. The exact mechanism of CRSwNP is still unknown; however, one of the hypotheses suggests that impaired epithelial cells function and eosinophilic mucosal inflammation are pivotal in nasal polyps development [6]. This process is followed by damaging factors, including bacterial biofilm, viruses, allergens, toxic substances or hypoxia resulting from existing airflow barriers (e.g., deviated nasal septum, concha bullosa) [7]. Moreover, hypersensitivity to aspirin and other Non-Steroidal Anti-Inflammatory Drugs (NSAIDs) is considered to be the causative agent of the disease. 

The prevalence of NSAIDs exacerbated respiratory disease (N-ERD) is about 10–20% among patients with CRSwNP [8,9]. Respiratory symptoms have been reported by 1.8% of the general European population, by 10–20% of patients with asthma [10,11], and upper airway disease in N-ERD patients is usually CRSwNP. On average, in N-ERD than in NSAIDs-tolerant CRSwNP patients, upper respiratory disease occurs as CRSwNP with worse symptoms, extent of sinuses opacification on CT scan and polyposis recurrence after surgery is observed [8,12,13]. Patients with N-ERD had undergone more sinus surgeries [8,14] (even two-fold; *p* < 0.001) and were significantly younger at the time of first surgery comparing NSAIDs-tolerant CRSwNP patients [8]. The prevalence of respiratory reactions to NSAIDs was higher in CRS patients with asthma symptoms in last 12 months but was not associated with allergic rhinitis [8]. Moreover, the prevalence of self-reported aspirin sensitivity was 2.3 in the control group, 3.3 in CRSsNP patients and 9.6 in CRSwNP patients [15]. Moreover, the CRSsNP group was significantly more likely than controls to report exacerbation of symptom due to ingestion of higher potential dietary salicylate content food [16]. 

The most possible ethiopathogenic theory of aspirin hypersensitivity seems to be cyclooxygenic. Cyclooxygenases are a group of key enzymes with a highly functional relevance mostly in the inflammatory process and cause the transformation of arachidonic acid leading to the formation of prostanoids, i.e., prostaglandins, thromboxanes and prostacyclins [17]. There are two basic isoforms of cyclooxygenase enzyme: COX-1 (constitutive COX, prostaglandin G/H synthase1, prostaglandin-endoperoxide synthase 1, prostaglandin H2 synthase-1) encoded by *PTGS1* gene located on the long arm of 9 chromosome consisting of 11 exons; and COX-2 (inducible COX, prostaglandin G/H synthase-2, prostaglandin-endoperoxide synthase-2, prostaglandin H2 synthase-2) encoded by a 10-exon *PTGS2* gene located on the long arm of 1 chromosome. The *PTGS1* gene has several characteristics sequences that are typical for housekeeping genes, e.g., no TATA sequence in the 5′ region or numerous transcription initiation sites. These locations contain many regulatory sequences that bind transcription factors such as: NF-κB, AP-2, SP-1 [18]. The 3′ end contains only one Show-Kamen sequence (ATTTA) responsible for reduced mRNA stability [19,20]. The *PTGS2* gene at the end of 5′ contains the classic TATA sequence located 31 nucleotides above the transcription site [21] and regulatory sequences binding transcription factors (NF-IL-6, C/WBP, AP-2, NF-κB, CRE, SP-1 and others) [22]. Their activation under the influence of growth factors, cytokines or stress is responsible for gene expression, which in turn can be inhibited by glucocorticosteroids and proinflammatory cytokines such as IL-4 or IL-10. De novo transcription of *PTGS2* gene is very fast in response to mitogens such as IL-1β, forbol esters and very similar to the activation of so-called early genes such as c-phos, c-jun and c-myc [21,22].

Aspirin, by acetylating serine in position 530 of the cyclooxygenase-1 amino acid chain, causes an irreversible change in enzyme protein conformation. The changed spatial structure of the active center prevents interaction of the enzyme with the arachidonic acid molecule. As a consequence, the synthesis of prostanoids, i.e., arachidonic acid metabolites of the cyclooxygenase pathway: prostaglandins and thromboxanes is inhibited [23]. Despite blocking the cyclooxygenase pathway, arachidonic acid remains a source of eicosanoids formed by lipoxygenases and cytochrome P450. According to the cyclooxygenase theory of aspirin hypersensitivity, cyclooxygenase inhibition results in an imbalance between the synthesis of the eicosanoids of the cyclooxygenase pathway with a diastolic effect on the smooth muscle of the bronchi (PGE2), and the synthesis of the lipoxygenase pathway eicosanoids causing bronchospasm (15-HETE, LTB4, cysteine leukotrienes), in favor of the latter [23]. Dysregulation of eicosanoid synthesis and an increased leukotriene production that is further accentuated by NSAIDs as cyclooxygenase-1 inhibitors, leads to a chronic and eosinophilic inflammation of the nasal and sinuses mucous membrane.

Cyclooxygenase-1 and -2 vary in susceptibility to aspirin inhibition due to different structure of the active center. The presence of valine instead of isoleucine in COX-2 at position 523 of the amino acid chain makes the active center channel of this enzyme isoform wider and its acetylation only changes the specificity of the reaction [23]. Bronchospasm attacks in asthmatic patients with aspirin hypersensitivity are caused by COX-1 blocking, while selective COX-2 inhibitors are well tolerated by them [24]. 

As aspirin causes a non-allergic hypersensitivity reaction only in people who are hypersensitive to ASA (acetylsalicylic acid), it is suggested that there are some initial, probably genetic differences in arachidonic acid metabolism in this group of patients [25]. Moreover, the search for factors influencing the development of CRSwNP with or without hypersensitivity to aspirin, as well as the advancement of molecular research have led to an interest in the phenomenon of alternative gene splicing, which is important for the diversity of proteins in human organisms. In the translation process preparation, the precursor messenger RNA (pre-mRNA) is edited in splicing by removing introns and joining exons together. Additionally, the alternative splicing may occur, resulting in creating a number of different mRNAs which are transcript variants of the same gene. If the mRNA splice variants relate to the coding sequence, the proteins differ in amino acid sequence, which may cause, e.g., differentiation of their function or location in the cell. Alternative splicing of non-coding regions may affect the presence of regulatory elements in mRNA, e.g., translational enhancer sequences or sequences affecting mRNA stability, and thus affect the amount of protein produced by the cell. Its regulation depends on the type of tissue and pathophysiological process coexistence [26]. Splicing dysregulation may lead to the disproportion of cyclooxygenase protein products transcript variants which could correlate with CRSwNP development. The knowledge about the role of *PTGS1* and *PTGS2* genes transcript variants in patients with the pathobiology of CRSwNP is still scarce. 

The main objectives of the work were identification of known and new, so far undescribed in human, transcriptional variants of *PTGS1* and *PTGS2* genes in CRSwNP patients and in the control group of healthy individuals; analysis of examined isoforms expressions in aspirin tolerant and intolerant patients with CRSwNP; determination of the relationship between the expression of transcript variant of *PTGS1* and *PTGS2* gene and the clinical course of CRSwNP. 

## 2. Materials and Methods

### 2.1. Study Groups

The study was performed on 409 adult patients: 206 patients suffering from CRSwNP and 203 healthy volunteers in the control group, all treated in Department of Otolaryngology, Head and Neck Oncology, Medical University of Lodz, Poland. All subjects gave their informed consent for inclusion before they participated in the study. The study was conducted in accordance with the Declaration of Helsinki, and the protocol was approved by the Bioethics Committee of Medical University of Lodz, Poland (RNN/187/18 July 2013/KE). 

The patients in the study group with CRSwNP underwent functional endoscopic sinus surgery (FESS) performed between 2014–2017 with following observation. Patients with contraindications to surgery, any history of cancer or immunological disorders were excluded from the study group. Diagnosis of asthma and the disease control were ascertained using GINA 2014 update [27]. The diagnosis of aspirin hypersensitivity was confirmed in Department of Internal Medicine, Asthma and Allergy by oral aspirin challenge as previously described [28].

Patients in the control group were hospitalized for planned surgeries not linked to chronic inflammatory or neoplastic diseases (e.g., rhinoseptoplasty, reposition of nasal bones after trauma). Patients with acute or chronic inflammation in head and neck region, a history of: cancer, immune system disorders, asthma, other lung diseases, allergy, atopic dermatitis, aspirin or other NSAIDs sensitivity were excluded from the control group. Positive family history of asthma or atopy and pathologic changes in chest X-ray were also exclusion criteria for the control group. 

The age of patients, duration of the disease (the patient’s age at the time of diagnosis), severity of clinical symptoms according to the VAS scale, severity of the disease in endoscopic nasal examination and in the computed tomography of the paranasal sinuses were analyzed. Patients with CRSwNP were asked to complete a questionnaire about the occurrence and duration of symptoms, which were assessed on a visual analog scale from 0 to 10, where “0” indicated no symptom presence and “10” signified the most severe nasal blockage/obstruction/congestion or nasal discharge (anterior/posterior nasal drip), facial pain/pressure and reduction or loss of smell. The score was evaluated before surgery and a validation study has shown mild disease to be defined as a VAS score of 0–3 inclusive, moderate as >3–7 inclusive, and severe as ≥7 [29]. Nasal endoscopy was performed to grade polyps extent on the basis of a 3-point classification system (0-absence of polyps; 1-polyps in middle meatus only; 2-polyps beyond middle meatus but not blocking the nose completely; 3-polyps completely obstructing the nose) [30]. The CT staging was assessed with the Lund–Mackay system which ranges from “0” as complete lucency of all sinuses to “24” with complete opacity of all sinuses [30]. A mild disease was defined as a CT score of 0–7 inclusive, moderate from 8–15, and severe from 16–24.

### 2.2. Blood and Tissue Samples

The biological materials collected from peripheral blood mononuclear cells (PBMC) from healthy volunteers (control group; N = 203) and tissue material of nasal polyps from operated patients, archived in containers filled with RNA-later solution (N = 206) were used for the RNA isolation. The isolation of the cells was carried out in a density-strict gradient according to the standard protocol. Modified Chomczyński method was used for RNA isolation from PBMC [31]. TRIreagent (Invitrogen, Carlsbad, CA, USA) was used for proper isolation from tumors and the process was carried out according to a standard protocol [32]. Total RNA was eluted in nuclease-free water and stored at −70 °C. The concentration of the RNA obtained was measured using a NanoDrop ND1000 sensitive spectrometer (Thermo Fisher Scientific, Waltham, MA, USA). The reverse transcription reaction was carried out using a commercially available High Capacity cDNA Archive Kit (Applied Biosystems, Foster City, CA, USA), and the obtained cDNA was brought to a concentration of 5 ng/μL and formed a matrix in further experiments.

### 2.3. Bioinformatical Analysis

Using the bioinformatic analysis and generally available medical databases, known variants (NM_080591, NM_000962, NM_000963) and new variants of *PTGS1* and *PTGS2* genes (NM_001271165.1, NM_001271368.1, NM_001271166.1, AY_151286, BQ_722004), previously unreported and hypothetically present in human were identified. The transcripts characteristics are available on the Human Genome Project website, University of California Santa Cruz Genome Browser GRCh38/hg38 [33]. It appeared that variants NM_001271165.1, NM_001271368.1, NM_001271166.1 are present in humans. 

We focused on *PTGS1* gene transcript variant named as NM_000962 (transcript variant 1; isoform 1) which contains a large exon 9 and is called a classic one. Another one examined was NM_080591 (transcript variant 2; isoform 2), and protein transcribed by this variant is slightly shorter. Additionally, we assumed the hypothetical occurrence of another transcriptional variant named as NM_001271165.1 (transcript variant 6; isoform 4). The next examined transcript variants were: NM_001271368.1 (transcript variant 7; isoform 6), which does not remove the previous exons but only the translation frame shift; and NM_001271166.1 (transcript variant 5; isoform 5), which causes the reading frame to move. In relation to *PTGS2* gene, we focused on one known transcript variant designated in genetic nomenclature as NM_000963.3 and two hypothetically existing in human named as: AY_151286 (isoform COX2b) and BQ_722004. We have presented the localization of investigated transcript variant in supplementary materials (Figure S1).

Primer Express (Applied Biosystems, Foster City, CA, USA) was used to design primers for each of the new transcripts, allowing amplification of them in PCR and assessment of specific expression in real time qPCR (primers presented in supplementary materials Table S1). Consequently it allowed identifying and distinguishing the contribution of each transcript in the disease process. The analysis of *PTGS1* and *PTGS2* expression was made using SYBR Green I fluorescence (Thermo Fisher Scientific, Waltham, MA, USA) and normalized with respect to human beta-actin expression. That enabled the determination of the absolute and reliable level of mRNA expression of *PTGS1* and *PTGS2* variants as previously was described [34]. Real-time analysis was performed in the 7900HT Real Time PCR genetic analyzer (Applied Biosystems, Foster City, CA, USA). The comparison of each variant of genes in individual patients was carried out using specialized computer programs SDS 2.3 and RQ 2.1 (Applied Biosystems, Foster City, CA, USA). Fold-change (FC) was calculated as 2^(-(dCtgroup2-dCtgroup1)) where dCt was calculated as the difference between the expression of the test gene and the reference gene. In the study, due to the availability of different biological material in study and control groups, the average dCT in each aspirin-resistant and aspirin-sensitive subjects was calculated, FC was calculated as 2^(-(dCtgroup2-dCtgroup1)) [35].

### 2.4. Analysis of the Coding Region of PTGS1 and PTGS2 Genes by Direct cDNA Sequencing

The PCR product obtained in the cyclic polymerase reaction (95 °C for 15 min followed by 40 cycles at 95 °C for 5 s, 60 °C for 30 s and 72 °C for 15 s; BioRad thermocycler T−100—BioRad, Hercules, CA, USA) with a set of specific pre-designed primer pairs encompassing the 3′ and 5′ UTR coding regions and 3‘and 5′ promoter regions was purified by column method (Qiagen, Hilden, Germany) providing a template for further analysis. Direct sequencing was performed using fluorescent labeled dideoxy-terminating nucleotides using one oligonucleotide primer designed for each transcript (transcript variant 1,2,4,5,6 of *PTGS1* and one transcript variant of *PTGS2*) as previously described [36]. The obtained mixture was purified by precipitation in ethanol with the addition of EDTA, and then suspended in deionized formamide (PerkinElmer, Waltham, MA, USA) and subjected to capillary electrophoresis using the ABI-PRISM310 genetic analyzer (Applied Biosystems, Foster City, CA, USA). Computer analysis of the obtained chromatograms was carried out using the available DNA Sequencing Analysis Software (Applied Biosystems, Foster City, CA, USA). Sequencher version 4.1.4 software (Gene Code, Ann Arbor, MI, USA) was used for submission and comparative analysis of the tested sequences. For the analysis of *PTGS1* and *PTGS2* gene sequences, a system of specific four pairs of oligonucleotide primers that duplicate the sequences of each gene was designed.

### 2.5. Statistical Analysis

Statistical analysis of the results was performed using the Statistica 13.0 software (StatSoft, Tulsa, OK, USA). For the statistical development of test results for numeric variables, mean and median values were calculated as well as standard deviation and interquartile range. Normal distribution of variables was estimated using the Shapiro–Wilk test. The majority of variables did not show a normal distribution; therefore, the significance of differences between average values of features in different groups was assessed using the non-parametric tests: U-Mann–Whitney or Kruskal–Wallis ANOVA in case of a number of groups greater than two. Correlations were evaluated by means of Sperman’s R range correlation. Comparisons of nominal variables between two groups were carried out using the χ^2^ test, whereas continuous variables between the two groups were made with the t-student test. For larger number of groups, the analysis of variance and post-hoc Tukey’s test were used in the case of statistical significance. Qualitative data was compared with the Yates-corrected χ^2^ test or Fisher’s exact test depending on the size of the groups. 

HaploView 4.1 computer program was used to analyze genetic couplings between the studied transcriptional variants, and to calculate the strength of the compound with phenotypic traits [37]. The analysis of the impact of the transcriptional variant on the clinical course was performed by the analysis of variance and multiple regression. The value of *p* < 0.05 was assumed as the level of significance.

## 3. Results

### 3.1. Basic Study Population Analysis

The study group consisted of 121 (58.74%) males. In the control group, there were 132 (65.02%) men. In the study group, the positive result of provocative test with aspirin enabled to identify 44 (21.36%) patients with coexisting aspirin intolerance and 57 patients (27.67%) who presented bronchial asthma. Among them, aspirin hypersensitivity was confirmed in 35 (16.99%). Therefore, most of the patients with aspirin intolerance (79.55%) presented all symptoms of aspirin triad. The characteristics of study and control groups are presented in Table 1.

For the purpose of this study, the transcript variants of the *PTGS1* and *PTGS2* genes were named as follows: COX1.1 for NM_000962, COX1.2 for NM_080591, COX1.3 for NM_001271165.1, COX1.4 for NM_001271368.1, COX1.5 for NM_001271166.1, COX2.1 for NM_000963.3, COX2.2 for AY_151286, and COX2.3 for BQ_722004. Transcripts were evaluated in various tissues (plasma and polyp tissue); therefore, a relative study of their expression was performed and assigned to ranks from the lowest to the highest for each patient. The rank “1” thus meant the smallest expression and the rank “5” the highest expression of the given transcript. Then, for each of the transcripts, the modal rank was counted and compared between the control and the study group.

The coexistence of all examined transcript variants of *PTGS1* and *PTGS2* genes in both the study and the control group was found and proved. The levels of each transcript variant expression of the *PTGS1* and *PTGS2* genes significantly differed in the study and the control group. Frequency of *PTGS1* and *PTGS2* isoforms for both groups are presented in Table 2.

### 3.2. Analysis of the Expression Level of PTGS1 and PTGS2 Genes Isoforms

**Figure 1:**
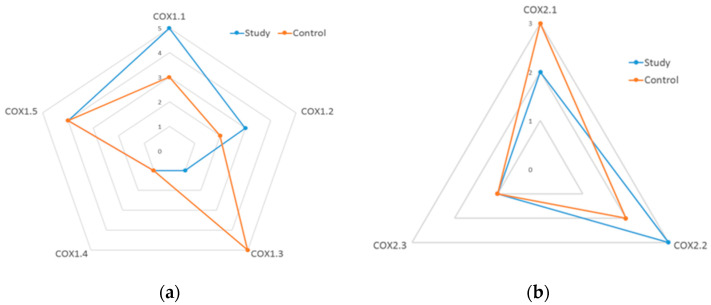
Comparison of *PTGS1* (**a**) and *PTGS2* (**b**) genes transcript variants expression in the study (N = 206) and control (N = 203) groups of patients. The graphs reveal the most common expression profile of transcript variant (the more outside the graph, the more often the specific variant in a given group was observed). COX1.1-COX1.5 and COX2.1-COX2.3 refers to transcript variants of *PTGS1* and *PTGS2* genes.

The comparison of modal levels of expression of individual transcript variants of *PTGS1* and *PTGS2* genes for the study and control groups is presented in Figure 1. The graphs show which transcript variants occurred more often in both groups and reveals the most common expression profile of transcript variants. The expression levels of the COX1.1 isoform significantly differed between both groups. In the study group, the most frequent was the strongest expression (rank 5) in comparison to the control group, where it was with the third expression (*p* < 0.001). The COX1.2 variant in the study group occurred with mean expression on rank 3. In the control group, the expression was lower, mostly at the level of 2nd and 1st rank (*p* < 0.0001). There were the greatest differences in COX1.3 variant between both groups. This isoform of the *PTGS1* gene was shown to be highly expressed in the control group, whereas low expression was observed in the study group (*p* < 0.0001). On the other hand, COX1.4 variant had similar expression in the control and the study group, with the prevalence of low ranks in the group of healthy volunteers (*p* < 0.0001). Expression of the COX1.5 transcriptional variant was high in both groups at rank level 4 with no significant differences in transcript expression between the study and the control groups (Table 2, Figure 1).

In the case of the COX2.1 isoform, lower values of expression measured as rank, were significantly more often found in the study group (*p* < 0.0001). The COX2.2 variant occurred with similar expression in both groups with higher expression in the study group (*p* = 0.0033). Comparing the expression of the COX2.3 variant in both groups, a significantly higher expression was observed in the study group (*p* < 0.0001). 

To sum up, it can be noticed that the expression ratio of individual variants was different in patients in the study and the control groups. The COX1.3 variant, which had the largest expression in healthy subjects, was replaced by COX1.1 and COX1.2 isoforms with a deflation of COX1.4 variant in the study group (*p* < 0.0005). In the *PTGS2* gene transcript variants, the expression of the most common COX2.1 isoform in the control group was reduced, while the expression of COX2.2 variant was increased in patients with CRSwNP. The expression of variant COX2.3 was low, both in the study and the control group. 

### 3.3. Expression Level of PTGS1 and PTGS2 Genes Transcript Variants and Aspirin Hypersensitivity

The expressions of all examined transcript variants were higher in aspirin hypersensitive CRSwNP patients in comparison to aspirin tolerant individuals. Although, some correlations were significant. In aspirin hypersensitive patients, the level of COX1.2 (NM_080591), COX1.3 (NM_001271165.1), COX1.4 (NM_001271368.1) and COX1.5 (NM_001271166.1) variants expression was significantly higher compared to aspirin-tolerant patients (Table 3). No such dependence was found for the *PTGS2* gene.

### 3.4. Analysis of Expression Levels of PTGS1 and PTGS2 Genes Transcript Variants in the Context of Clinical Data

**Figure 2:**
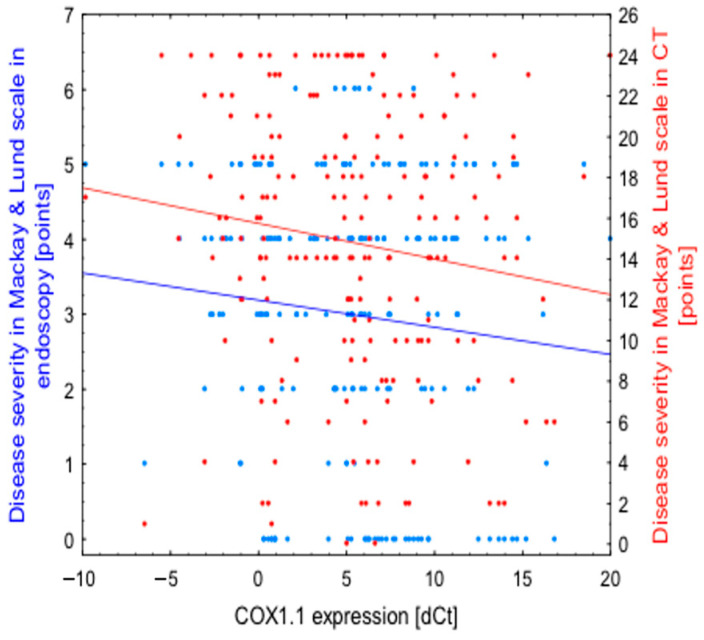
Correlations between expression of COX1.1 transcript variant and disease severity. Higher dCt values represent lower expression, hence a negative correlation with dCt represents a positive association between expression of a given variant and disease severity.

In further studies, no differences in transcript variants expression levels were found between the patients in the study and the control groups according to patient’s age at the time of the study, age at onset of illness, gender and comorbidities including bronchial asthma. However, correlations between expression of COX1.1 transcript variant and disease severity were found (Figure 2, Table 4). The severity of symptoms assessed by the VAS scale before surgery was higher in patients with increased expression of variants: COX1.1 (R = −0.134; *p* = 0.0490), COX1.3 (R = −0.1429; *p* = 0.0400) and COX1.5 (R = −0.1499; *p* = 0.032). In the COX2.1 variant, the relationship was close to statistical significance (R = −0.1173; *p* = 0.0940). The expression of the COX1.1 isoform (R = −0.098; *p* = 0.049) and COX1.5 (R = −0.141; *p* = 0.043) also increased with greater extent of polyps in endoscopic examination. Moreover, with the increase in the severity of changes measured as sinuses opacity in computed tomography, the expression of the COX1.1 variant significantly increased (R = −0.163; *p* = 0.020). In addition, the COX2.3 variant expression increased; however, this relationship was close to statistical significance (R = −0.118; *p* = 0.095). There were no similar relationships between the remaining variants of both the *PTGS1* and *PTGS2* genes and the advancement of CRSwNP in endoscopy or computed tomography. 

## 4. Discussion

The rapid development of molecular researches has recently led to the search for genetic markers of predisposition to the development of CRSwNP, also in patients with or without NSAIDs hypersensitivity. To the best of our knowledge, no studies have been conducted to identify transcriptional variants of *PTGS1* and *PTGS2* genes present in these patients so far. In this study, using bioinformatic analysis and generally available medical databases, several and potentially new, previously undescribed forms of these genes were identified in the *PTGS1* and *PTGS2* genes region. We confirmed the co-occurrence of all the above transcriptional variants both in patients with CRSwNP and the control group. 

The results of our study have shown significant differences in the expression of the transcript variants of *PTGS1* and *PTGS2* genes while comparing patients with CRSwNP to the control group. The detailed analysis showed that in the case of *PTGS1* gene, the replacement of COX1.3 (NM_001271165.1), which had typically the highest expression in healthy people, with COX1.1 (NM_000962) and COX1.2 (NM_080591) transcripts with a slight decrease of COX1.4 isoform (NM_001271368.1) in patients with CRSwNP was observed. The transcripts of COX1.1 (NM_000962) and COX1.2 (NM_080591), appearing in much higher concentrations in humans with CRSwNP, seem to be activated by ongoing chronic inflammation. The variants COX1.1 and COX1.2 differ from each other in the length of the exon 9, which in the case of the variant COX1.2 (NM_080591) is shorter. This results in the synthesis of a smaller protein based on the COX1.2 mRNA matrix, consisting of 562 amino acids. Expressions of COX1.1 (NM_000962) and COX1.2 (NM_080591) variants differed among themselves, with the prevalence of COX1.1 form over COX1.2 in both the control and the study group. In contrast to these isoforms, the COX1.3 variant with the highest expression in the control group, resulting from the activation of an additional exon within intron 2, designated as 3a and being the focus area of the EST sequences, encodes a much smaller protein than the classic variant, consisting of only 490 amino acids. This protein does not contain the first two potential glycosylation areas (Asn 67, Asn 103) involved in the formation of a tertiary structure and the coupling of the enzyme to the cell membrane. In the most recent update of the human Hg38 genome, the function of the protein encoded by this isoform as being compatible with the COX-1 enzyme function was evaluated. Potentially, a protein based on the mRNA of the COX1.3 transcriptional variant (NM_001271165.1) may have less activity than the basic form consisting of 599 amino acids. The decrease in expression of this variant might indicate its protective role in the process of CRSwNP development.

Variant COX1.4 (NM_001271368.1) has an additional exon within intron 2. Unlike the COX1.3 isoform, this does not remove the previous exons but only causes the translation frame shift. The total number of exons of this isoform is 12, with 10 exons being coding. The resulting protein contains 537 amino acids. Decreased expression of this variant in patients with CRSwNP might be a cause of reduced activity of encoded protein, similarly as in the case of the COX1.3 isoform. 

The expression of the next tested COX1.5 variant (NM_001271166.1) was quite high in both groups without any significant differences between the control and the study group. This isoform is characterized by the occurrence of a longer exon 1, which in turn causes a shift of the reading frame. The variant consists of a total of 11 exons, with 7 exons coding. The encoded protein is much shorter and consists of only 453 amino acids. The protein product and the variant itself are the least characterized compared to the other tested isoform of *PTGS1* gene.

In the case of the *PTGS2* gene, it was noticed that the expression of the most common COX2.1 isoform (NM_000963.3) in the control group was reduced in favor of increased expression of the COX2.2 isoform (AY_151286) in patients with CRSwNP. The expression of the COX2.3 isoform (BQ_722004) did not differ significantly between patients in the study and the control group. There were no significant correlations between *PTGS2* gene isoforms and sensitivity to aspirin, although the expression levels of examined isoforms were slightly higher in aspirin intolerant patients in comparison to aspirin tolerant individuals. They do not seem to be relevant to the promotion of an aspirin intolerant CRSwNP.

The analysis of the presence of alternative products of the *PTGS1* gene and their influence on the regulation of transcriptions was for the first time conducted by Diaz et al. [38]. Cyclooxygenase-1 has four potential areas of glycosylation, these are amino acids: Asn 67, Asn 103, Asn 143 and Asn 409. They are believed to be involved in the formation of the tertiary structure and the bonding of the enzyme to the cell membrane. Deletion of 111 nucleotides in exon 9 eliminates the glycosylation site of Asn 409. Synthesized protein deprived of 37 cut amino acids may have a different, significantly reduced enzymatic function or lose it completely. The reduction of cyclooxygenase-1 enzymatic activity may cause a decrease in prostaglandin (PGE2) synthesis, which in turn may upset the balance between the synthesis of eicosanoids with diastolic effect and eicosanoids constricting the bronchial smooth muscle. Variable expression of this transcript after activation of TNFβ, IL-1β, TNFα and phorbol esters was demonstrated in human fibroblasts [38]. Studies have also shown that this transcription variant is more frequent in patients with asthma compared to healthy individuals and correlates with 15-HETE secretion after aspirin stimulation [9]. There were statistically significant differences between the expression of that variant called in our study as COX1.2 (NM_080591) and hypersensitivity to aspirin. It was confirmed that in aspirin intolerant patients coexisting with CRSwNP, the expression of variant COX1.2 was higher than in patients with aspirin tolerance. Also, expressions of COX1.3, COX1.4 and COX1.5 variants were significantly higher in aspirin intolerant patients. This may confirm the contribution of these isoforms to the promotion of the CRSwNP in aspirin hypersensitive patients. However, this should still be confirmed by further studies. 

In rat tracheal cells, a *PTGS1* gene (used to be called *COX-1*) transcript variant that lacks exon 1 but instead contains part of intron 2 was identified. Giving that exon 1 contains a translational initiation codon, this transcript was suspected to encode a nonsense protein [1]. Nevertheless, Vogiagis et al. [39] showed that the expression of this variant in rats increased in colon tumors, and decreased after treatment with NSAIDs as compared to healthy individuals. The authors [39], when conducting research on this alternative form of splicing, described it as a variant of COX-1SV, formed on the *PTGS1* (*COX-1*) RNA matrix, but missing the first 150 nucleotides. It is known that prostaglandins, which are products of cyclooxygenase metabolism, have an important role in the defense of the stomach mucosa. Vogiagis et al. [40] showed changes in the expression of the COX-1SV variant compared to the classic isoform depending on the age. The COX-1SV isoform was dominant in the elderly; therefore, it was postulated that it may have a significant relation with the decreasing mucosal protection. Vogiagis et al. found in subsequent studies that COX-1SV is 2% of the total COX-1 protein mRNA expression in rat colorectal cancer cells. They showed that the level of COX-1SV increased in the case of tumors of the large intestine and rectum and then decreased, after the use of non-steroidal anti-inflammatory drugs to the values detected in the mucosa of healthy rats [39]. In this study, there were no statistically significant differences in the expression of that protein named COX 1.3 variant depending on the age of the patients. However, a significant correlation was observed between the expression of COX1.3 form and the presence of CRSwNP: In patients in the control group, the expression was significantly higher compared to the patients in the study group. Moreover, the significant reduction of COX1.3 and COX1.4 expression levels in patients with aspirin tolerance compared to the patients with intolerance to aspirin, where these levels were already lower in the whole study group than in the control group, may be important in the development of chronic rhinosinusitis in the patients with hypersensitivity to aspirin. The lower expression levels of COX1.3 and COX1.4 variants may be related to lesser severity of disease and decrease the likelihood of a recurrence in this group of patients.

Chandrasekharan et al. in dogs’ brains identified another *PTGS1* gene transcript, which contains intron 1, as an insertion inside an open reading frame, resulting in encoding additional 30 amino acids, called signal peptide. This transcript was named COX-3. The protein thus created is characterized by reduced prostaglandin synthesis ability and specific sensitivity to acetaminophen [41]. In humans, it is likely that additional mechanisms of alternative folding, shifting the ribosome reading frame or so called RNA editing may also lead to this transcript and altered protein [41]. This hypothesis seems all the more credible as three sizes of mRNAs for *PTGS1* gene were found in humans: 2.8-kb, 4.5-kb and 5.2-kb [42]. The 2.8-kb transcript is the most frequently detected and probably encoding the primary protein form for *PTGS1*. The 4.5-kb transcript is poorly characterized. However, the 5.2-kb variant found in humans in the cerebral cortex, heart, muscles, as well as bladder and colon may contain intron-1 and correspond to COX-3 in dogs [41]. In further studies, it was found that COX-3 is not encoded by a separate gene, but it is a transcript variant of the *PTGS1* (“COX-1”) gene [43]. Instead of the name “COX-3”, the use of the “COX-1b” name was suggested, and the expression of COX-1b mRNA was found in many human organs, including the heart, brain, stomach and liver [44,45]. 

Chandrasekharan et al. also characterized another variant of the alternative *PTGS1* (COX-1) gene: PCOX-1, or so called partial COX-1, caused by the deletion of exons 5–8, which causes the loss of 219 amino acids from the catalytically active site, both from the cyclooxygenase and prostaglandin domain. This resulted in losing the ability of the enzyme to synthesize prostaglandins, with the ability to oxidize or isomerase fatty acids preserved [41]. Two forms of PCOX-1 have been identified: PCOX-1a, additionally contains intron 1, as well as COX-3, while PCOX-2b does not contain this additional insertion [41]. The structure of the COX-1 and COX-2 genes in all mammals is strictly conservative, with the exception of the intron 1. The exons of both these genes may be deleted inside the reading frame, resulting in new proteins with altered enzymatic function.

There is only single report concerning *PTGS2* gene and transcript variants. Evanson, in his studies, was interested in the *PTGS2* gene for cyclooxygenase-2 and observed COX-2 mRNA in various organisms as three alternative transcripts: 4.2-, 3.8- and 2.2-kb. He postulated that 3.8-kb and 2.2-kb transcripts are the result of the polyadenylation of non-coding areas containing AUUAAA sequences [46]. No studies have been conducted on the expression of *PTGS2* transcript variants in humans yet.

Our results carry a number of clinical implications. Namely, there were significant correlations suggesting the link between expression of COX1.1 transcript variant and disease severity. We observed that the results in VAS scale, related to the reported symptoms before surgery, were bigger in patients with higher expression of COX1.1, COX1.3 and COX1.5 variants. The expression of COX1.1 and COX1.5 isoforms also increased with advancement of severity of polypoid lesions in endoscopic examination. Additionally, with the extension of changes in computed tomography, the expression of the COX1.1 variant significantly raised. Therefore, the COX1.1 isoform was significantly more frequent in cases with higher clinical advancement. No such dependence was observed for any other variants of *PTGS1* and also for *PTGS2* genes’ investigated isoforms. Potentially, further research in these fields may result in the development of a more individualized treatment approaches. 

## 5. Conclusions

To sum up, it should be noticed that the expression of the studied transcriptional variants of the *PTGS1* and *PTGS2* genes is different in patients with CRSwNP compared to the control group. Moreover, in the development of the phenotype of CRSwNP, both in patients without and with hypersensitive to aspirin, known and new variants of *PTGS1* and *PTGS2* genes are involved and importasnt. The variants COX1.1 (NM_000962), COX1.2 (NM_080591) and COX2.2 (AY_151286) are not only more common in patients compared to the controls but are also prevalent in cases with less favorable CRSwNP; thus, it seems that they may be involved in the promotion of a more aggressive disease. From the other hand, the transcriptional variants COX1.3 (NM_001271165.1), COX1.4 (NM_001271368.1) and COX1.5 (NM_001271166.1) of the *PTGS1* gene and the transcript COX2.1 (NM_000963.3) of the *PTGS2* gene were observed in CRSwNP patients with milder course of CRSwNP. Participation of the examined variants in occurring CRSwNP in aspirin intolerant patients is not certain. 

## Figures and Tables

**Table 1:** Characteristics of the study (N = 206) and the control (N = 203) groups. (R: right nasal cavity; L: left nasal cavity).

Phenotypic Trait	Study Group						Control Group
	All CRSwNP		Aspirin Tolerance		Aspirin Intolerance		
Number (%)	206 (100)		162 (78.64)		44 (21.36)		203 (100)
Females N (%)	85 (41.26)		62 (38.27)		23 (52.27)		71 (34.98)
Males N (%)	121 (58.74)		100 (61.73)		21 (47.73)		132 (65.02)
Mean age (range)	46.1 (18–79)		46.29 (18–79)		45.20 (20–71)		41.72 (18–65)
Mean age of CRSwNP diagnosis (range)	39.79 (9–75)		40.47 (9–75)		36.49 (12–66)		
Bronchial asthma N (%)	57 (27.67)		22 (10.68)		35 (16.99)		
Mean number of surgeries (range)	1.40 (1–10)		1.18 (1–7)		2.89 (1–10)		
Mean preoperative VAS scale (SD)	4.4 (2.2)		4.5 (2.4)		4.2 (2.0)		
Endoscopical extent of polyposis N (%):	R	L	R	L	R	L	
No polyps	45 (21.84)	54 (26.21)	42 (25.93)	50 (30.86)	3(6.82)	4 (9.10)	
1	25 (12.14)	24 (11.70)	22 (13.58)	20 (12.35)	3 (6.82)	4 (9.10)	
2	113 (54.85)	89 (43.20)	81 (50.00)	66 (40.74)	32 (72.73)	23 (52.27)	
3	23 (11.17)	39 (18.93)	17 (10.49	26 (16.05)	6 (13.63)	13 (29.54)	
Mean Lund-Mackay CT score (SD)	16.5 (6.5)		14.9 (4.7)		19.8 (6.2)		

**Table 2:** The transcript variants of the *PTGS1* and *PTGS2* genes along with the ranks of frequency of occurrence in the study (N = 206) and the control (N = 203) groups. (std—standard deviation).

Type of Variant	Group	Mean rank of Expression (Range, std.)	Median	Modal
COX1.1	Study group	4.48 (1.00–5.00; 1.09)	5.00	5.00
COX1.2		2.88 (1.00–5.00; 0.98)	3.00	3.00
COX1.3		1.85 (1.00–5.00; 0.87)	2.00	1.00
COX1.4		2.25 (1.00–5.00; 1.27)	2.00	1.00
COX1.5		3.45 (1.00–5.00; 1.05)	4.00	4.00
COX 2.1		2.23 (1.00–3.00; 0.67)	2.00	2.00
COX 2.2		2.38 (1.00–3.00; 0.65)	2.00	3.00
COX 2.3		1.27 (1.00–3.00; 0.59)	1.00	1.00
COX1.1	Control group	3.78 (1.00–5.00; 0.95)	4.00	3.00
COX1.2		1.82 (1.00–4.00; 0.58)	2.00	2.00
COX1.3		4.16 (2.00–5.00; 0.87)	4.00	5.00
COX1.4		1.41 (1.00–5.00; 0.83)	1.00	1.00
COX1.5		3.78 (1.00–5.00; 0.87)	4.00	4.00
COX 2.1		2.54 (1.00–3.00; 0.54)	3.00	3.00
COX 2.2		2.39 (1.00–3.00; 0.54)	2.00	2.00
COX 2.3		1.07 (1.00–3.00; 0.34)	1.00	1.00

**Table 3:** Expression of individual transcriptional variants of *PTGS1* and *PTGS2* genes depending on the presence (N = 44) or lack of hypersensitivity to aspirin (N = 162) in the study group (N = 206). Higher dCt values represent lower variant expression. (FC—fold-change).

Type of Variant	Aspirin Tolerance Mean dCT (Range, std.)	Aspirin Intolerance Mean dCT (range, std.)	FC	*p*
COX1.1	5.81 (0.98–9.07; 5.52)	4.88 (0.13–10.58; 5.81)	1.91	0.318
COX1.2	10.43 (8.87–12.17; 4.45)	9.37 (8.03–10.91; 4.30)	2.08	0.034
COX1.3	11.57 (9.66–13.41; 4.63)	9.90 (7.12–12.07; 4.30)	3.18	0.003
COX1.4	11.31 (7.76–14.99; 5.62)	9.59 (6.74–12.28; 5.14)	3.29	0.049
COX1.5	8.81 (6.83–11.04; 4.20)	7.70 (5.71–9.69; 3.66)	2.16	0.021
COX2.1	6.54 (3.96–8.38; 4.33)	5.32 (1.94–7.90; 3.56)	2.33	0.124
COX2.2	6.50 (4.10–8.04; 4.90)	5.45 (2.52–7.82; 3.82)	2.07	0.294
COX2.3	10.66 (6.16–15.07; 6.46)	9.64 (5.20–13.66; 6.00)	2.03	0.348

**Table 4:** Correlations between expression of specific *PTGS1* and *PTGS2* genes transcription variants and disease severity determined by the endoscopic evaluation and computed tomography (CT). Higher dCt values represent lower expression, hence a negative correlation with dCt represents a positive association between expression of a given variant and disease severity.

Expression (in dCt)	Disease Severity in Endoscopic Evaluation	Disease Severity in Lund-Mackay CT Scale
COX1.1	r = −0.1103	r = -0.1462
	*p* = 0.118	*p* = 0.038
COX1.2	r = −0.0194	r = −0.0552
	*p* = 0.785	*p* = 0.436
COX1.3	r = −0.0196	r = −0.0009
	*p* = 0.781	*p* = 0.990
COX1.4	r = −0.0016	r = −0.0887
	*p* = 0.982	*p* = 0.210
COX1.5	r = −0.0839	r = −0.0579
	*p* = 0.234	*p* = 0.412
COX2.1	r = −0.0612	r = −0.1065
	*p* = 0.385	*p* = 0.130
COX2.2	r = −0.0376	r = −0.0458
	*p* = 0.613	*p* = 0.537
COX2.3	r = −0.0863	r = −0.1063
	*p* = 0.227	*p* = 0.136

## Data Availability

All subjects gave their informed consent for inclusion before they participated in the study. The study was conducted in accordance with the Declaration of Helsinki, and the protocol was approved by the Ethics Committee of Medical University of Lodz, Poland (RNN/187/18 July 2013/KE).

## Supplementary Materials

The following are available online at https://www.mdpi.com/2075-4418/11/1/135/s1. Table S1: Primers for amplification of the different transcriptional variants of the *PTGS1* and *PTGS2* genes used in the study. Figure S1: Localization of the transcript variant of *PTGS1* (A) and *PTGS2* (B) genes investigated in the study.

## Abbreviations

ASA	acetylsalicylic acid
COX-1	cyclooxygenase-1 (constitutive COX, prostaglandin G/H synthase1, prostaglandin-endoperoxide synthase 1, prostaglandin H2 synthase-1)
COX-2	cyclooxygenase-2 (inducible COX, prostaglandin G/H synthase-2, prostaglandin-endoperoxide synthase-2, prostaglandin H2 synthase-2)
CRSsNP	chronic rhinosinusitis without nasal polys
CRSwNP	chronic rhinosinusitis with nasal polyps
CT	computed tomography
FESS	functional endoscopic sinus surgery
NSAIDs	non-steroidal anti-inflammatory drugs
N-ERD	non-steroidal anti-inflammatory drug (NSAIDs)-exacerbated respiratory disease
*PTGS1* gene	prostaglandin-endoperoxide synthase 1 gene
*PTGS2* gene	prostaglandin-endoperoxide synthase 2 gene
VAS	visual analogue scale
