# Supplementary material for: Expression of Transcript Variants of PTGS1 and PTGS2 Genes among Patients with Chronic Rhinosinusitis with Nasal Polyps
Source: Diagnostics (Basel). 2021 Jan 16;11(1):135. doi: 10.3390/diagnostics11010135 (PMC7830232; doi:10.3390/diagnostics11010135)
Supplement: Supplementary file 1 [file diagnostics-11-00135-s001.pdf]

Supplementary Table S1. Primers for amplification of the different transcriptional variants of the *PTGS1* and *PTGS2* genes used in the study.

Gene	Transcript variants	Primers
PTGS1	COX 1.1	F: CAACCGTGTGTGTGACCTGCTG<br>R: GCAATCTGGCGAGAGAAGGCATC
	COX 1.2	F: CAACCGTGTGTGTGACCTGCTG<br>R: CCCCACCGATCTTGAAGGAGTC
	COX 1.3	F: CAGCTGTTGAGGGCCTGGAAG<br>R: GTCACACTGGTAGCGGTCAAGG
	COX 1.4	F: GGTTCTTGCTGTTCCTGCTCCTG<br>R: CAGCGAGGCAACCTGAAGAGC
	COX 1.5	F: GGGGCAGGGTATGTGGTTCAG<br>R: GAGCAGGACGGGGAGCGG
PTGS2	COX 2.1	F: GCCTGAGTATCTTTGACTGTGGGAG<br>R: GTCAAAGCACTTCACGCATCAG
	COX 2.2	F: CATCCCCTTCTGCCTGACACCTTTC<br>R: TGCTCTGGTCAATGGAAGCCTGTG
	COX 2.3	F: CCGCTGGGAGTTTCAGTTTTACCT<br>R: CTACCAGAAGGGCAGGATACAGC

A.

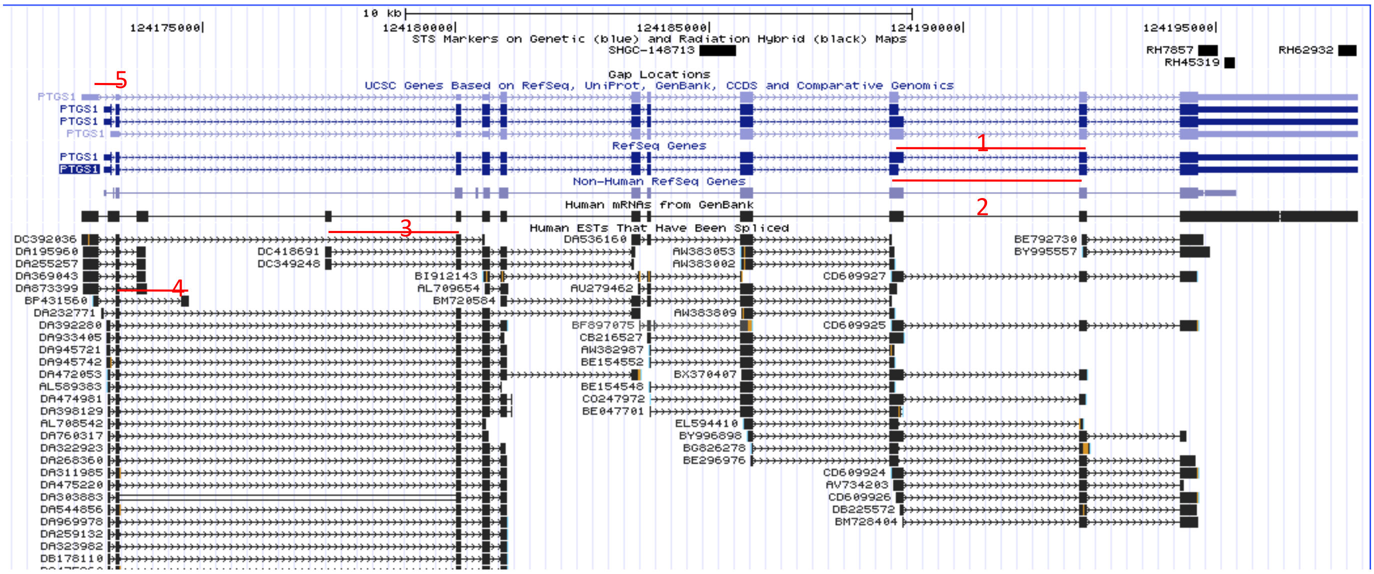

B.

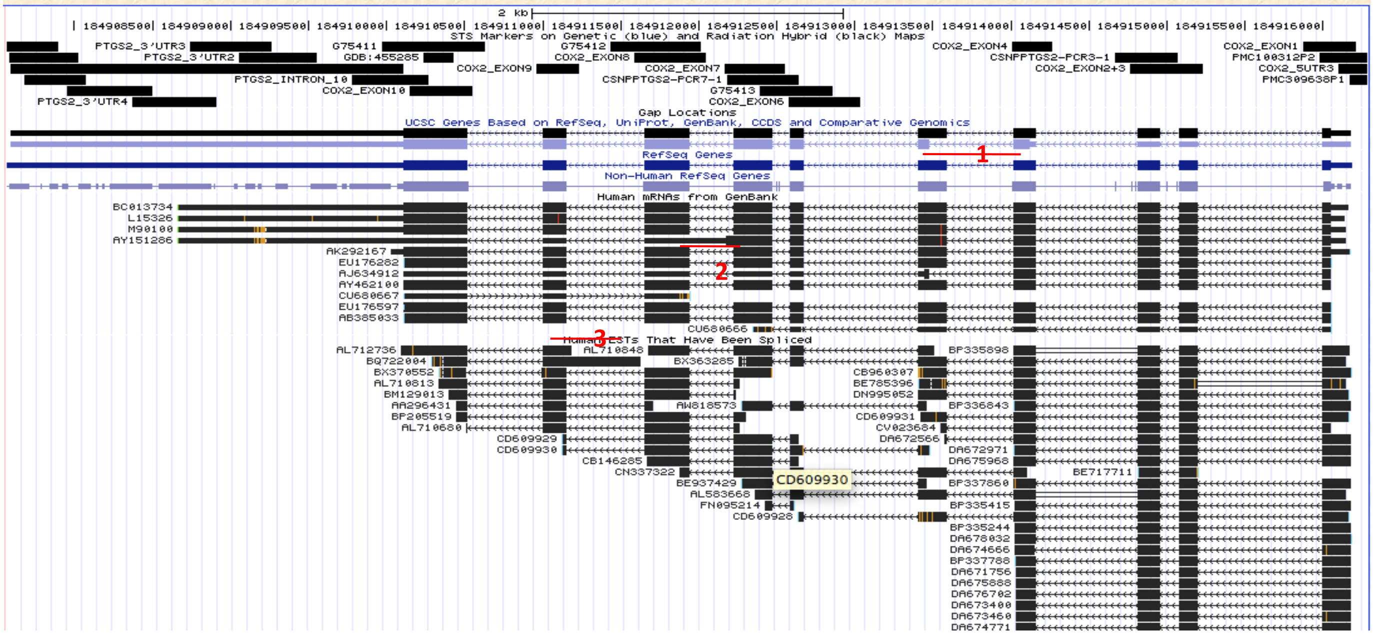

Supplementary Figure S1. Localization of the transcript variant of *PTGS1* (A) and *PTGS2* (B) genes investigated in the study.
